# Recurring violence against adolescents: an analysis of notifications*

**DOI:** 10.1590/1518-8345.6277.3681

**Published:** 2022-10-03

**Authors:** Franciéle Marabotti Costa Leite, Isaura Barros Alves Pinto, Mayara Alves Luis, José Henrique Iltchenco, Mariana Rabello Laignier, Luís Carlos Lopes-Júnior

**Affiliations:** 1 Universidade Federal do Espírito Santo, Departamento de Enfermagem, Centro de Ciências da Saúde, Vitória, ES, Brasil.

**Keywords:** Adolescent, Violence, Exposure to Violence, Recurrence, Health Information Systems, Nursing

## Abstract

**Objective:**

to identify the frequency of reported cases of recurring violence against adolescents and their association with victim, violence, and aggressor characteristics.

**Method:**

cross-sectional study conducted with notified data on violence against adolescents, produced by Epidemiological Surveillance and registered in the Information System of Diseases and Notification (SINAN), from 2011 to 2018, in the state of Espírito Santo, Brazil.

**Results:**

the frequency of recurring violence against adolescents was 46.4%. Higher occurrence was observed among girls (PR: 1.26; 95%CI: 1.15 - 1.38) between 10 and 14 years of age (PR: 1.20; 95%CI: 1.13 - 1.28), and people with a disability or disorder (PR: 1.52; 95%CI: 1.42 - 1.62). Psychological violence/neglect was 30% more prevalent in recurrence than self-harm. Most cases occurred at home (PR: 1.56; 95%CI: 1.37 - 1.77). Results showed a 1.11 times higher prevalence of recurring violence perpetrated by aggressors aged 20 years or older and higher evidence in male aggressors (95%CI: 0.97 - 1.17).

**Conclusion:**

recurring violence was associated with victim, aggressor, and event characteristics. Health intersectoriality is crucial to reduce cases of recurring violence.

## Highlights

(1) Frequency of recurring violence against adolescents was 46.4%.

(2) Higher recurrence was observed among girls between 10 and 14 years of age.

(3) Most cases occurred at home.

(4) Recurring violence was associated with victim, aggressor, and event characteristics.

(5) Results show 1.11 times > prevalence of recurring violence perpetrated by men ≥ 20 years old.

## Introduction

Violence against adolescents manifests itself in many ways, whether perpetrated by parents, other caregivers, colleagues or strangers in this life cycle, individuals are exposed to self-harm; domestic abuse and neglect by parents and caregivers; to youth violence outside the home, often in schools, in the community, or online; and intimate partner violence or dating violence, which occurs among adolescents involved in romantic relationships^1^.

Regardless of its form, violence is a traumatic event that has negative individual, social, and economic impact^2^. Depending on its occurrence - chronic or recurrent -, consequences can be dire. One of the most disturbing aspects of child abuse is its tendency to recur^3^. The literature points out that recurrence of violence refers to circumstances in which victims who have previously been proven victims of abuse or neglect experience another incident of proven abuse^4^.

In 2017, Brazil recorded 79,914 reports of violence against adolescents, of which 30.9% were recurring cases and most were perpetrated in domestic settings. The Southeast region, in the same year, recorded 36,521 cases of violence, of which 29.5% were repeat cases^5^.

Recurrence has multiple and significant negative effects on children and adolescents who are abuse victims^4^. Regarding sexual violence, one study concluded that those who experience a childhood episode of sexual abuse exhibit more risky sexual behaviors and are more likely to experience further episodes of sexual victimization during adolescence and early youth^6^. Moreover, adolescent victims of recurrent violence tend to experience more episodes of violence at school, more aggression in the community, and further transgress social norms. They also have less social support, lower resilience and low self-esteem^7^ ^-^ ^8^.

Recurrence of abuse is of great interest for protection services to victims of violence^3^, context in which health professionals, especially nurses, play an important role in identifying cases of acute or chronic violence for better referral of victims in the health care network, aiming at resoluteness and comprehensive care. This identification by nurses also provides subsidies for organization and performance of the social protection network for adolescents^9^. Besides, case reporting contributes to the epidemiological dimensioning of the issue, which allows apprehending the dynamics of this violence, and then developing and creating specific programs and actions, as well as public policies aimed at its prevention^10^.

Given this context, this study sought to identify the frequency of reported cases of recurring violence against adolescents and their association with victim, violence, and aggressor characteristics.

## Method

### Study design

This is a cross-sectional observational analytic study^11^.

### Location

The study took place in the state of Espírito Santo (ES), Brazil, one of the federation units that make up the Southeast region and has a territorial area of 46,074,447 km². According to the last census of the Brazilian Institute of Geography and Statistics (IBGE) in 2010, its Human Development Index (HDI) corresponds to 0.740, thus having the 7^th^ best HDI among the Brazilian states. Adolescent inhabitants between the ages of 10 and 19 totaled 603,898,000 people in the last census.

### Population and study period

The research was conducted with notified data on violence against adolescents, between 10 and 19 years of age, produced by the Epidemiological Surveillance and registered in the Information System of Diseases and Notification (SINAN), from 2011 to 2018, in the state of Espírito Santo.

The World Health Organization (WHO) defines adolescence as the period of life beginning at age 10 and ending at age 19. For the WHO, adolescence is divided into three phases: early (10-13 years), middle (14-16 years) and late (17-19 years) adolescence^12^.

### Study variables

Dependent variable was recurring violence (yes or no). Independent variables included victim characteristics: gender (male and female), age group (10-14 years and 15-19 years), ethnicity/color (white and non-white), disability/disorder (yes or no) and area of residence (urban and rural/peri-urban); event characteristics: type of violence (physical, sexual, psychological/neglect, self-harm and other) and place of occurrence (home, public street and other); and aggressor characteristics: number of aggressors involved (one and two or more), age of the aggressor (up to 19 years and 20 years or older), gender of the aggressor (male, female and both), relation to the victim (family, stranger and other/acquaintance), and aggressor suspected of alcohol use (yes or no).

### Data analysis

Data were analyzed using descriptive statistics in raw and relative frequency with 95% confidence intervals. Bivariate analyses were performed using Chi-Square test (χ²), with a significance level of 95%, and adjusted analysis using Poisson’s regression with robust variance. All independent variables with p< 0.20 were included in the model. Permanence occurred when p < 0.05.

### Ethical aspects

Study approved by the Research Ethics Committee of the Universidade Federal do Espírito Santo, under opinion no. 2,819,597. All the standards and guidelines of Resolution 466/2012 were followed.

## Results

From 2011 to 2018, the state of Espírito Santo recorded a total of 3,094 cases of recurring violence against adolescents, that is, a frequency of 46.4%.

Most adolescent victims of recurring violence were girls (78.3%), between 15 to 19 years old (52.5%), non-white (72%), with no disability and/or disorder (80.7%) and living in the urban area (90.7%). Physical violence (34%) was the most frequent type of abuse, and recurring violence mostly happened at home (79.3%) (Table 1).

**Table 1:** Characterization of adolescent victims of recurring violence (N=3,094). Vitória, ES, Brazil, 2011 to 2018

Variables	Recurring violence		
	n	(%)	95%CI*
Gender			
Male	672	21.7	20.30 - 23.20
Female	2,422	78.3	76.79 - 79.69
Age group (years)			
10 to 14	1,469	47.4	45.72 - 49.24
15 to 19	1,625	52.5	
Ethnicity/Color			
White	791	28.0	26.35 - 29.66
Non-white	2,036	72.0	70.33 - 73.64
Disability/Disorder			
No	2,238	80.7	79.22 - 82.16
Yes	534	19.3	17.83 - 20.77
Area of residence			
Urban	2,801	90.7	89.66 - 91.70
Rural/Peri-urban	286	9.3	8.29 - 10.34
Type of violence			
Physical violence	1,052	34.0	32.35 - 35.69
Sexual violence	811	26.2	24.69 - 27.79
Psychological violence/Neglect	197	6.4	5.55 - 7.28
Self-harm	1,005	32.5	30.85 - 34.15
Other	29	0.9	0.65 - 1.34
Place of occurrence			
Home	2,297	79.3	77.74 - 80.69
Public street	354	12.2	11.07 - 13.45
Other	247	8.5	7.55 - 9.59
Number of aggressors involved			
One	2,601	86.3	84.99 - 87.45
Two or more	414	13.7	12.54 - 15.00
Age of aggressor (years)			
Up to 19 years old	1,298	55.6	53.54 - 57.57
20 or older	1,038	44.4	42.42 - 46.45
Gender of the aggressor			
Female	1,112	37.3	35.54 - 39.01
Male	1,724	57.8	55.99 - 59.53
All genders	148	5.0	4.23 - 5.79
Relation to the victim			
Family	985	48.0	45.86 - 50.19
Strangers	693	33.8	31.77 - 35.86
Other/acquaintances	373	18.2	16.57 - 19.91
Aggressor suspected of alcohol use			
No	1,648	76.5	74.70 - 78.28
Yes	505	23.5	21.71 - 25.94

*CI = Confidence Interval

Data showed a higher frequency of violence perpetrated by a single person (86.3%), mainly male (57.8%), up to 19 years old (55.6%). In 48% of the cases, the aggressor was a family member. In most cases (76.5%), the aggressors were not suspected of having consumed alcohol before the assault (Table 1).

The bivariate analyses (Table 2) showed that the distribution of the event characteristics was associated with the variables gender, age group, ethnicity/color, disability/disorder, type of violence, place of occurrence, number of aggressors involved, gender of the aggressor, and relation to the victim (p < 0.05).

**Table 2:** Distribution of characteristics of recurring violence notifications among adolescents (N=3,094). Vitória, ES, Brazil, 2011 to 2018

Variables	Recurring violence			
	n	(%)	95%CI*	p^†^
Gender				
Male	672	34.9	32.82 - 37.08	0.000
Female	2,422	51.0	49.63 - 52.47	
Age group (years)				
10 to 14	1,469	53.3	51.45 - 55.17	0.000
15 to 19	1,625	41.5	39.99 - 43.08	
Ethnicity/Color				
White	791	49.0	46.60 - 51.48	0.003
Non-white	2,036	45.9	44.43 - 74.36	
Disability/Disorder				
No	2,238	43.1	41.77 - 44.46	0.000
Yes	534	70.4	67.09 - 73.59	
Area of residence				
Urban	2,801	46.4	45.17 - 47.69	0.801
Rural/Peri-urban	286	45.9	42.02 - 49.84	
Type of violence				
Physical violence	1,052	36.8	35.05 - 38.59	0.000
Sexual violence	811	50.0	47.59 - 52.43	
Psychological violence/Neglect	197	74.6	69.01 - 79.51	
Self-harm	1,005	53.4	51.14 - 55.64	
Other	29	67.4	52.06 - 79.79	
Place of occurrence				
Home	2,297	57.0	55.43 - 58.49	0.000
Public street	354	23.6	21.53 - 25.83	
Other	247	33.3	30.02 - 36.21	
Number of aggressors involved				
One	2,601	48.9	47.53 - 50.21	0.002
Two or more	414	43.4	40.27 - 46.56	
Age of aggressor (years)				
Up to 19 years old	1,298	50.5	48.53 - 52.39	0.060
20 or older	1,038	53.3	51.06 - 55.49	
Gender of the aggressor				
Female	1,112	51.8	49.67 - 53.90	0.000
Male	1,724	44.4	42.84 - 45.96	
All genders	148	71.8	65.30 - 77.57	
Relation to the victim				
Family	985	41.2	39.27 - 43.21	0.000
Strangers	693	65.9	63.01 - 68.74	
Other/acquaintances	373	32.1	29.52 - 34.90	
Aggressor suspected of alcohol use				
No	1,648	49.0	47.27 - 50.65	0.606
Yes	505	48.05	45.03 - 51.07	

*CI = Confidence Interval; †p = p-value

In examining the adjusted analysis (Table 3), we note a 1.26 times higher prevalence of recurring violence in girls compared to boys (PR: 1.26; 95%CI: 1.15 - 1.38). Adolescents aged 10 to 14 years had a 20% higher prevalence than those age in the 15-19 years’ group (PR: 1.20; 95%CI: 1.13 - 1.28). Those who had some type of disability and/or disorder were 52% more likely to be victimized when compared with the group without these conditions (PR: 1.52; 95%CI: 1.42 - 1.62).

**Table 3:** Raw and adjusted effects analysis of the characteristics of cases of recurring violence among adolescents (N=3,094). Vitória, ES, Brazil, 2011 to 2018

Variables	Raw analysis			Adjusted analysis		
	PR*	95%CI^†^	p^‡^	PR*	95%CI^†^	p^‡^
Gender						
Male	1.0	--	0.000	1.0	--	0.000
Female	1.46	1.36 - 1.56		1.26	1.15 - 1.38	
Age group (years)						
10 to 14	1.28	1.22 - 1.35	0.000	1.20	1.13 - 1.28	0.000
15 to 19	1.0	--		1.0	--	
Ethnicity/Color						
White	0.93	0.88 - 0.99	0.028	0.95	0.88 - 1.01	0.157
Non-white	1.0			1.0	--	
Disability/Disorder						
No	1.0	--	0.000	1.0	--	0.000
Yes	1.63	1.54 - 1.72		1.52	1.42 - 1.62	
Area of residence						
Urban	1.01	0.92 - 1.10	0.802	1.05	0.95 - 1.16	0.294
Rural/Peri-urban	1.0	--		1.0	--	
Type of violence						
Physical violence	0.68	0.64 - 0.73	0.000	0.96	0.84 - 1.09	0.000
Sexual violence	0.93	0.87 - 0.99		1.00	0.87 - 1.16	
Psychological violence/Neglect	1.39	1.28 - 1.51		1.30	1.11 - 1.52	
Other	1.26	1.02 - 1.56		1.57	1.09 - 2.25	
Self-harm	1.0	--		1.0	--	
Place of occurrence						
Home	1.70	1.53 - 1.89	0.000	1.56	1.37 - 1.77	0.000
Public street	0.70	0.61 - 0.81		0.75	0.62 - 0.89	
Other	1.0	--		1.0	--	
Number of individuals involved						
One	1.12	1.04 - 1.21	0.003	1.11	1.00 - 1.22	0.090
Two or more	1.0	--		1.0	--	
Age of aggressor (years)						
Up to 19 years old	1.0	--	0.059	1.0	--	0.036
20 or older	1.05	0.99 - 1.11		1.11	1.00 - 1.23	
Gender of the aggressor						
Female	1.0	--	0.000	1.0	--	0.000
Male	0.85	0.81 - 0.90		1.06	0.97 - 1.17	
All genders	1.38	1.26 - 1.52		1.37	1.20 - 1.57	
Aggressor suspected of alcohol use						
No	1.0	--	0.608	1.0	--	0.690
Yes	0.98	0.91 - 1.05		0.98	0.89 - 1.07	

*PR = Prevalence Ratio; †CI = Confidence Interval; ‡p = p-value

Psychological violence/neglect and other types of violence, respectively, 30% and 57% more prevalent in recurring abuse than self-harm (PR: 1.30; 95%CI: 1.11 - 1.52 and PR: 1.57; 95%CI: 1.09 - 2.25). Moreover, most cases of recurring violence took place in the victim’s home (PR: 1.56; 95%IC: 1.37 - 1.77).

Regarding aggressor characteristics, there is an 11% higher prevalence of recurring violence perpetrated by aggressors aged 20 years or older compared to the up-to-19 years group (RP: 1.11; 95%CI: 1.00 - 1.23). Regarding the gender of the aggressor, the variable “both genders” was more prevalent when compared to male and female assailants individually (PR: 1.37; 95%CI: 1.20 - 1.57).

## Discussion

This study sought to understand the context of recurring violence against adolescents according to victim, event, and aggressor characteristics. We found that recurring violence against adolescents in the state of Espírito Santo between 2011 and 2018 showed a frequency of 46.4%. A nationwide study with data reporting violence against adolescents registered in SINAN between 2011 and 2017 observed that 39.9% of the events were recurring cases^13^.

Regarding victim characteristics, we observed a higher prevalence of cases of recurring violence among female adolescents (PR: 1.26; 95%CI: 1.15 - 1.38). This finding is similar to a study carried out based on notifications to the Guardianship Councils and care programs in the city of Londrina, Paraná, from 2002 to 2006, where most cases of recurring violence reported occurred among female victims (66.3%)^14^. Another research states that women were victims of recurring violence to a greater extent than men^13^.

This higher prevalence of recurring violence among women can be explained by historical and cultural factors, which imposed conditions of abuse, exploitation, subordination and discrimination on women, resulting in current gender issues that place girls at higher risk of exposure to violence and its recurrence^15^ ^-^ ^16^.

Adolescent girls bring with them a movement of freedom and search to overcome femininity stereotypes. International literature has been reporting the developmental perspective related to gender and violence within intimate relationships^17^. Intimate relationships usually begin in adolescence, and their knowledge concerning these relationships is tied to the media, as well as to the observation of friends and family. Such a process is permeated by narcissism, an attachment to gender-specific roles, and the mystification of romantic love, leaving this population vulnerable to intimate partner abuse^17^. This perspective is also addressed by a meta-analysis on the prevalence of violence in intimate physical and sexual relationships among adolescents, and its associated factors^18^.

As for age group, we note a higher prevalence of recurring violence among younger adolescents (10 to 14 years old) (PR: 1.20; 95%CI: 1.13 - 1.28). A study conducted with secondary data from Sample II of the National School Health Survey (PeNSE 2015), pointed out that among adolescent victims of intrafamily violence, those in the youngest age group (13 to 15 years old) had higher prevalence of recurring violence (P: 8.6%. 95%CI: 7.6 - 9.6) when compared with older adolescents^19^.

The higher number of cases of violence against younger age groups is justified due to their inability to escape and/or defend themselves. Moreover, physical and personality fragility makes adolescents easy targets of violence and contributes to the chronicity of such abuse^14^ ^,^ ^20^.

Younger adolescents are physically, psychologically and socially more vulnerable, lacking maturity to understand the web created by the aggressor to exert violence^21^. Besides, they are more likely to experience violence because they spend more time at home with their families when compared to older age groups, who generally spend more time outside their homes^19^.

International studies have addressed adolescents living in high vulnerability urban regions, and point to statistically significant differences in the understanding and coping of this vulnerable population regarding violence in intimate relationships^18^ ^,^ ^22^. The context of social vulnerability can expose adolescents to situations of violence with negative repercussions for their lives. A systematic review that summarized and evaluated data on the causes and consequences of dating violence among adolescents, pointed out that poverty is an important factor for its occurrence^23^. It also suggested that financial hardship and low income also increase the risk of violence in intimate relationships^24^.

A qualitative study that aimed to understand and analyze the perceptions of highly vulnerable adolescents regarding the construction of violent intimate relationships, identified that traditional gender norms still play a key role in shaping adolescent relationships. Such behaviors are more visible in dating relationships, when commitment and exclusivity are seen as main characteristics; furthermore, jealousy emerged as the main trigger of violence, and technologies were the main contemporary tools to reinforce it^25^.

Another finding of the present study was the higher prevalence of recurring psychological violence/neglect among adolescents (PR: 1.30; 95%CI: 1.11 - 1.52). A nationwide study conducted between 2011 and 2017 found that recurring violence was associated with a higher occurrence of psychological violence and neglect^13^. Both are difficult to register due to their characteristic subjectivity and different expressions, being generally associated with other forms of violence^26^ ^-^ ^27^.

In the present study, the higher prevalence of recidivism of violent behavior was associated with having some type of disability and/or disorder (PR: 1.52; 95%CI: 1.42 - 1.62). Data similar to the research conducted by the U.S. company Westat, which showed that violence against adolescents with disabilities and/or disorders occurred 1.7 times more often than among adolescents without disabilities^28^. Certain characteristics of certain types of disabilities, such as communication difficulties, lack of knowledge on how to defend oneself, poor social skills, and excessive dependence on others, can facilitate situations of violence and the chronicity of these events^29^ ^-^ ^31^. Interestingly, studies on recurring violence among people with disabilities or disorders is scarce in the literature, demonstrating the need for further scientific research on this subject.

Most cases of recurring violence took place at the victim’s home (PR: 1.56; 95%CI: 1.37 - 1.77). This is in line with a study^32^ conducted with data from the Violence and Accidents Surveillance System (VIVA) - which makes up the Sinan - in the state of São Paulo, in 2009, which founded that 72.9% of the cases of violence against adolescents happened in domestic settings. Another research^13^ showed that about 57% of the cases took place at home, pointing to this space as a common place for violence to emerge.

The victim’s home is seen as a privileged environment for the occurrence of multiple episodes of violence against adolescents, since the limits imposed by the physical and social privacy of the environment isolate the family from the public eye, providing a place without witnesses and covered up by family complicity^14^.

Regarding the aggressors’ characteristics, we observed a higher prevalence of recurring violence perpetuated by individuals aged 20 years or older (PR: 1.11; 95%CI: 1.00 - 1.23). This data corroborates a study conducted with cases of recurring violence reported to the Sinan, in the state of Espírito Santo, from 2011 to 2018, showing that the practice of recurrent violence predominated among individuals aged 20 years or older (78.5%) when compared to individuals aged between 0 and 19 years^33^.

Students in different countries have ratified the importance of developing preventive actions and programs to peers and bystanders regarding violence against children and adolescents^34^ ^-^ ^36^. These programs have significantly reduced the perpetration of and victimization by intimate partner violence^35^ ^-^ ^36^. A study conducted in schools in Pennsylvania, USA, identified that students were enthusiastic about discussing healthy and unhealthy relationships with school nurses. Many adolescents subjected to dating violence have reported their experience to these nurses.

One limitation of the present study concerns the lack of database completeness for some independent variables. Importantly, the material available in the literature on recurring violence is scarce and mostly focuses on recurrent violence against women and/or children under the age of 10. Hence, we highlight the importance of this research for contributing to a bibliographic collection on the subject, and in encouraging further research on the same topic to broaden knowledge and shed light on new ways of addressing the core issue - recurring violence against adolescents.

## Conclusion

In conclusion, the study found a high prevalence of recurring violence against adolescents. The highest prevalence of this event occurred among girls, between 10 and 14 years of age, who had some type of disability and/or disorder. Psychological violence/neglect were the most frequent and committed by aggressors 20 years and older of all genders.

Given these findings, we must invest in professional training to track and address this type of violence, as well as to promote multidisciplinary humanized care and break the cycle of violence against adolescents. This serious public health issue needs to be addressed, ensuring these individuals a healthy and safe development. We highlight the importance of joint actions between healthcare, public sectors, and civil society to reduce cases of recurring violence.

Furthermore, is of utmost importance to notify and qualify databases, since knowing epidemiological data about violence against adolescents can contribute to the dimensioning of this issue and consequently to the formulation of protection strategies for this group.
